# Potato peel extracts as an antimicrobial and potential antioxidant in active edible film

**DOI:** 10.1002/fsn3.1119

**Published:** 2020-11-13

**Authors:** Haftom Y. Gebrechristos, Xiaochi Ma, Fang Xiao, Yonghuan He, Shuwen Zheng, Gurjav Oyungerel, Weihua Chen

**Affiliations:** ^1^ Institute of Food Science and Technology CAAS Beijing China

**Keywords:** antimicrobial, antioxidant, edible film, potato peel

## Abstract

Potato peel is phenolic‐rich plant byproduct that has multiple biological functions. The study explores the antimicrobial efficiency of the peel and its antioxidant potential incorporated with potato starch film. Antimicrobial sensitivity test using microdilution and diffusion test revealed positive response in *Escherichia coli*, *Salmonella enterica*, and *Staphylococcus aureus* with minimum inhibitory concentration, 7.5 ± 2, 5.8 ± 2, and 4.7 ± 1 mg/ml, respectively, but had a negative response for *Klebsiella pneumoniae* and *Listeria monocytogenes*. HPLC analysis revealed that *caffeic, chlorogenic,* and *neochlorogenic* acids are the main chemical compounds found in potato peel extract and responsible for its antimicrobial property. The scavenging ability and phenolic content in the active film range from 10 to 22 mg GAE/g of the dried film with 24%–55% of inhibition, respectively. The scanning electron microscope of the starch film showed homogeneously and ordered structure; however, some cracks and pores shown in the active film. The simulation experiment of phenols migration from the film into fatty and aqueous foods showed higher concentration in aqueous and less in the fatty foods. Therefore, potato peel extract has antibacterial as well as antioxidant property and incorporating with potato starch produce an active film which can be used as an alternative technology for active food packaging.

## Graphical abstract

Potato peel extract has antibacterial and antioxidant property and has excellent potential to be used as active edible film food packaging incorporating into potato starch film.

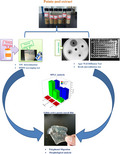

## 1 INTRODUCTION

Potato is the fourth main stable crop next to rice, wheat, and maize consumed widely all over the world. Nowadays, types of processed potato products have increased from time to time to meet the tremendous interest of consumers. Food processing factories produce many volumes of wastes as a byproduct (Charmley, Nelson, & Zvomuya, 2006; Schieber & Aranda, 2009). Peels of vegetables are the main byproduct of plant processing factories which has important organic compounds. Potato waste contains valuable chemical components like phenols which are suitable to apply in food preservation and pharmaceutical industries (Grunert, 2018; Crandall & Ricke, 2008).

Antimicrobials used to preserve packed foods are chemical compounds applied in or added to foods that inhibit or kill microbes. For decades, there was an interest to use plant origin natural antimicrobials/herbs/ as food preservation (Burt, 2004; Klančnik, 2010). The plant‐originated extracts offer unlimited opportunities for appropriate additives and drug treatments because of their diversified chemical property (Kujala, Pihlaja, Vuorela, & Vuorela, 2000).

Fat oxidation is the main factors influence the value of food because this reaction results in off‐flavor of processed foods (Kingston, Monahan, Buckley, & Lynch, 1998). Fat undertakes prominent oxidative changes at boiling temperature during storage; however, the addition of antioxidant delays the oxidation reaction. Potato peels (PP) is one of the phenolic‐rich food processing industrial wastes and has a strong antioxidant ability which is equal to the synthetic antioxidants like butylated hydroxyanisole (BHA) and butylated hydroxytoluene (BHT). Generally, in food processing industries, synthetic antioxidants are the most efficient ways to decrease rancidity and toxic oxidation molecules, but they have a long‐term consumer's health hazard (Gebrechristos & Chen, 2018; Ito et al., 1986; Perelman et al., 1998; Sébédioo, Kaitaranta, Grandgirarda, & Malkk, 1991). Such concerns and demand for safe food for consumers the food industry seek innovative food preservation strategies. The recent trends in food packaging technology are focusing on the development of films that play an effective role in the preservation of food quality (Galdi, Nicolais, Maio, & Incarnato, 2008). Therefore, to my knowledge, a study dealing with PP extracts incorporated with potato starch film to produce active film has not performed yet. So the objective is exploring the antimicrobial efficacy of PP extracts and its antioxidant potential incorporation with an active film made of potato starch.

## 2 MATERIAL AND METHOD

### 2.1 Standard chemicals and chemical reagents

Dried and milled potato peel (PP), potato starch, analytic grade glycerol (plasticizer), Sodium hydrogen carbonate (NaHCO_3_), aluminum chloride (AlCl_3_), 2,2‐diphenyl‐1‐picrylhydrazyl, Gallic acid, and Folin–Ciocalteu's phenol reagent purchased from Beijing chemical factory PLC.

### 2.2 Preparation of PP extracts

Fresh potato was purchased from local Market, washed, peeled, and dried in a hot air oven (Horizontal Forced Air Drier, Proctor and Schwartz Inc.) at 55°C. The dried PP was ground into a fine powder in a mill (Tecator Cemotec 1090 samples mill). The material that passed through an 80 mesh sieve was reserved for use. About 10 g of ground PP was extracted with 100 ml of ethanol overnight in a shaker at room temperature. The extract was filtered through cheesecloth, and the residue was re‐extracted three times under the same conditions. The combined filtrate was evaporated in a rotary evaporator (EVF‐530‐010K‐GallenKamp) below 40°C temperature. The extracted dried powder was then stored at −20°C.

### 2.3 Antimicrobial effect of PP

#### 2.3.1 Bacterial strains

A total of five bacterial strains attained from food science and technology institution, CAAS microbiology laboratory were used to test the antimicrobial effects of the PP extract. These include three strains of gram‐negative bacteria and two strains of gram‐positive bacteria (Table 1). All strains of these bacteria are well‐known food‐borne bacterial pathogens, except *E. coli* which used as surrogates for *E. coli* O157:H7. Before used, the bacterial strains were consecutively subcultured three times in 24 hr interval. From the subcultured bacteria strain, 10μl of culture transferred into 10 ml liquid medium, and then the media incubated at 37°C for 24 hr.

**Table 1:** Profile of tested bacteria

Microorganism	Gram reaction	ATCC	Culture media
*E. coli*	−	25922	Tryptic soy broth
*S. enterica*	−	1311	Nutrient broth
*K. pneumonia*	−	2473	Tryptic soy broth
*S. aureus*	+	6538	Tryptic soy broth
*L. monocytogenes*	+	19115	Brain heart infusion broth

Abbreviations: *E. coli*, *Escherichia coli*; *S. enterica*, *Salmonella enterica*; *K. pneumonia*, *Klebsiella pneumonia*; *S. aureus*, *Staphylococcus aureus*; *L. monocytogenes*, *Listeria monocytogenes*.

#### 2.3.2 Agar test for bacteria

The inhibitory effects of PP extract tested bacteria were determined by agar well diffusion method (Oke, Aslim, Ozturk, & Altundag, 2009). The autoclave media was added to 50°C in a water bath, and an overnight culture of the bacterial strain added at a final concentration of 10^5^ CFU/ml. The prepared suspension poured into a sterile Petri dish and kept for 1h at room temperature to solidify. A well (diameter = 6 mm) was prepared. PP extract was diluted to the concentration of 5 mg/ml with broth, and 50 μl of diluted extract was dispensed into each agar well. Plates were kept for at least 1 hr at room temperature to allow the extracts to diffuse into the agar before incubation at 37°C for 24 hr. After incubation, the degree of inhibition was expressed as clear (++), vague (+), and no inhibition zone (−). Only samples with a result of clear and vague inhibition zone were used to determine the minimum inhibitory concentration (MIC) and the minimum lethal concentration (MLC).

#### 2.3.3 MIC and MLC of PP extract

The MIC and MLC of PP extract performed using a broth microdilution method. Forty‐five well culture plates were prepared, and serial twofold dilutions of the extracts were distributed into the plate wells. The volume of dispensed extract was 0.1 ml per well in the concentration range of 10–0.31 mg/ml. The same volume (0.1 ml) of overnight bacterial culture at a density of 10^5^ CFU/ml was added to the wells, and the culture plates were placed in an incubator for 24 hr at 37°C.

MIC was selected as the lowest concentration of the PP extract required to inhibit the visible growth of the tested microorganism. The MLC was also determined by streaking the suspension in the well with concentrations greater than the MIC. After that, the subcultured agar plates were incubated overnight at 37°C. The MLC was defined as the lowest concentration of extract at which no viable microorganism was detected by subculture.

### 2.4 Antioxidant potential of PP in active edible film

#### 2.4.1 Aqueous potato peel extracts (PPE)

Aqueous potato peel extracts were prepared with 10 g of dried and milled potato peel mixed with 100 ml of distilled water and placed in a thermostatic bath at 50°C for 60 min. After obtained the mixed potato extracts were cooled, filtered (pore size 0.45 mm), and stored at 4°C in dark flasks until used. The extraction yield, as determined gravimetrically at 80°C until constant weigh, was 1 mg of dried extract/ml of sample. The solution was used in different proportion to make active edible film.

#### 2.4.2 Active edible film preparation

Potato starch film was prepared by mixing 93 g of distil water 5 g potato starch 1.5 g of glycerol. The mixture will start out white in color and change to transparent or translucent. It will also thicken. Once the initial white color of the starch is completely gone and the mixture has thickened remove from the heat. The thickened solution was poured into a labeled dish, the air bubbles removed by glass stir rod and then placed in a stable, labeled table for air dry. This film considered as control film (PSF) but the active films were prepared, a water mass (5, 10 or 20 g) from the formulations replaced with the same amount of aqueous PP extract so the active film referred as PSF‐PPE 5%, PSF‐PPE 10%, and PSF‐PPE 20%. Each blend homogenized for 40 min and then heated until 96°C (heating rate with 3°C/min), under constant stirring. The formulations were degassed for 7 min with a mechanical vacuum pump, dispensed into polypropylene plates, and dried at 50°C for 24 hr. Films were conditioned at room temperature into desiccators containing supersaturated solution of sodium bromide (RH = 57%) for 48 hr, prior to characterization studies.

#### 2.4.3 Film observation in SEM

The prepared starch film (refer Section 2.4.2) was cut in to 20 mm size manually using sharp scissors. Three pieces of each sample were immersed into 5 ml of supersaturated solution of sodium bromide. Micrographs of cross sections of the films were obtained using a Field emission scanning electron microscopy (FE‐SEM). The specimens were cryofractured by immersion in liquid nitrogen. The samples were mounted on stubs and sputtered with a thin layer of gold (thickness below 50 nm) prior to SEM observations. Images at different magnifications (2,000× up to 10,000×) were obtained using a voltage of about 3 kV and a spot size of ~2 nm. The thickness of each film was measured from SEM images at six randomly selected points, using the ImageJ software. Accordingly, selected images taken for analysis.

#### 2.4.4 Phenol content of PPE and active PSF

Extract of PP dilution in cleaned water numerous concentration levels. The composition of film extract mixed 80 mg of the test films with 5 ml distilled water (PSF‐PPE 5%, PSF‐PPE 10% and PSF‐PPE 20%). The mixtures sited 125 rpm shaker in a room for 24 hr, and the supernatant was taken examined by Folin–Ciocalteu method (Lamuela‐ravents, 1999). Total phenolic content (TPC) of PP determined with spectrophotometric. A methanolic solution of PP extract in 1 mg/ml concentration used for chemical analysis. The sample prepared 0.5 ml, methanol of PP extract 2.5 ml of Folin–Ciocalteu's reagent (10%) liquefied in water 2.5 ml and 7.5% NaHCO_3_. The blank solution prepared without PP extract. A mixture contains 0.5 ml methanol, 2.5 ml 10% Folin–Ciocalteu's reagent liquefied, and 2.5 ml of 7.5% of NaHCO_3_. The samples incubated at 45°C for 45 min. The absorbance determined using spectrophotometry at (*λ*_max_ = 760 nm) ready in triplicate and absorbance recorded for analysis. Through the gallic acid calibration standard curve, the TPC stated as mg of GA/g of PP extract.

The ability of PP extracts for free radicals scavenging assessed by DPPH assay. The sample methanol solution of PP extracts was ready in 1 mg/ml dilutions. The mixed 1 ml and with the equal amount of DPPH in methanol 1 mg/ml concentration and after 30 min in dark room temperature (23°C), the absorbance noted at 515 nm. The antioxidant ability tested as defined in 100 ml sample mixed with 3.9 ml of (DPPH) ethanol solution (25 mg DPPH/L) and observed at 515 nm until reaction created. The DPPH‐scavenging activity of each sample expressed as the inhibition percentage calculated with the following equation.

(1) $$\text{DPPH}\;\%\;\text{inhibition}=\left(\frac{\text{Ab}-\text{As}}{\text{Ab}}\right)\times 100$$

where Ab is the blank and As is the sample.

#### 2.4.5 Phenolic migration of PPE

Polyphenol migration tests were performed considering water and ethanol 95% as food simulators for aqueous and fatty foods, respectively (Baner, Bieber, Figge, Franz, & Piringer, 1992). Samples film pieces of 2 cm^2^ were immersed in 5 ml of food simulant and placed in a shaker at 25°C and 125 rpm for 7 days. After completing the exposure time, the migration of PP polyphenols to each simulant was tested by the Folin–Ciocalteu method. The sample prepared 0.5 ml of the simulant solution 2.5 ml of Folin–Ciocalteu's reagent (10%) liquefied in water 2.5 ml and 7.5% NaHCO_3_. The blank solution prepared without the simulant solution. A mixture contains 0.5 ml methanol, 2.5 ml 10% Folin–Ciocalteu's reagent liquefied, and 2.5 ml of 7.5% of NaHCO_3_. The samples incubated at 45°C for 45 min. The absorbance determined using spectrophotometry at (*λ*_max_ = 760 nm) ready in triplicate and absorbance recorded for analysis. Through the gallic acid calibration standard curve, the TPC stated as mg of GA/g of the simulant solution (Busolo & Lagaron, 2015).

#### 2.4.6 HPLC–DAD analysis of phenolic compounds

Phenolic acids of potato peel were purified by microporous membrane filtration and determined its content by high‐performance liquid chromatography using standard chemicals of chlorogenic acid standard, neochlorogenic acid, and caffeic acid. 5.00mg dissolved in 30% methanol. The standard stock solution was prepared at a concentration of 100 mg/ml and stored in 4°C in the dark part of the refrigerator. The phenols of potato peel extract were determined using high‐performance liquid chromatography with an external standard of chlorogenic acid, neochlorogenic acid and caffeic acid dissolved in 30% methanol at a concentration rate of 100 mg/ml. Column: YMC‐C18 column (250 mm × 4.6 mm, 5 μm); Mobile phase: methanol (A), 0.5% phosphoric acid solution (B) gradient elution (0–10 min: A 25%–40%; 10–60 min: A 40%–60%); Injection of 10 μl, column temperature of 25°C used. The standard curve built through the standard stock solution (4.6) with 30% methanol diluted with the concentration of 0.02, 0.10, 0.50, 1.00, 2.00 mg/L standard working solution, according to 6.3 determination The chlorogenic, neochlorogenic, and caffeic level were observed, and the corresponding integral peak area ratio was the ordinate, and the standard curve or linear regression equation drawn. To determine the corresponding standard working solution and the sample sampled in order to retain the time qualitatively, the chromatographic peak area integral amount, the sample solution of chlorogenic neochlorogenic, and caffeic acid response were within the range of quantitative determination.

The results calculated and expressed

(2) X=CV/M

where C—concentration of chlorogenic acid, neochlorogenic, and caffeic, mg/l:l, V—the final volume of the sample solution, ml; M—quantity of the sample, g.

## 3 RESULT AND DISCUSSION

### 3.1 Antimicrobial nature of PP

The antimicrobial activity of PP extracts was determined by agar well diffusion method, and the detail results are available in (Table 2). The sensitivity of the extract was found to differ significantly among the test organisms. The extract prevented the growth of *E. coli, S. enterica,* and *S. aureus* unlikely did not show a zone of inhibition on *K. pneumonia* and *L. monocytogenes.* For accurate determination of the antimicrobial activity, a microdilution assay was performed. The susceptibility of *E. coli, S. enterica,* and *S. aureus* against PP extract was evaluated, and the results are presented as MICs and MLCs (Table 2). The MIC/MLC value considering the 10 mg/ml treatment of PP extract was 7.5 ± 2, 5.8 ± 2, and 4.7 ± 1 mg/ml for *E. coli*, *S. enterica*, and *S. aureus,* respectively. Edible antimicrobial films are developed in order to reduce the growth of microorganisms on the surface of foods. Antimicrobial films have potential application as active packaging, and polyethylene antimicrobial films show inhibitory effects against *S. aureus* and *E. coli* (Geany, Camilloto, Fátima, Soares, & Clarissa, 2009; Wobeto & Andrade, 2006).

**Table 2:** Zone of inhibition and MIC or MLC of PP

Bacteria strains	ZoI	PPE (mg/ml)	*E. coli*	*S. aureus*	*S. enterica*
*E. coli*	+	5	+	+	+
*S. enterica*	++	2.5	−	+	+
*K. pneumonia*	−	1.25	−	+	−
*S. aureus*	++	0.63	−	−	−
*L. monocytogens*	−	0.31	−	−	−

Abbreviations: *E. coli*, *Escherichia coli*; *S. enterica*, *Salmonella enterica*; *K. pneumonia*, *Klebsiella pneumonia*; *S. aureus*, *Staphylococcus aureus*; *L. monocytogenes*, *Listeria monocytogenes*; MIC, minimum inhibition concentration; MLC, minimum lethal concentration; ZoI, zone of inhibition.

The result from agar well diffusion test and microdilution test proven that PP extracts excellent antimicrobial efficacy for both gram‐negative and gram‐positive microbes. However, it exhibited relatively higher antibacterial ability to hinder *S. aurues* growth. This suggests the inhibitory properties of PP extract for microbes were definite on gram‐positive than on gram‐negative bacteria. Similar results found in an earlier study by Amanpour (2015).

**Figure 1:**
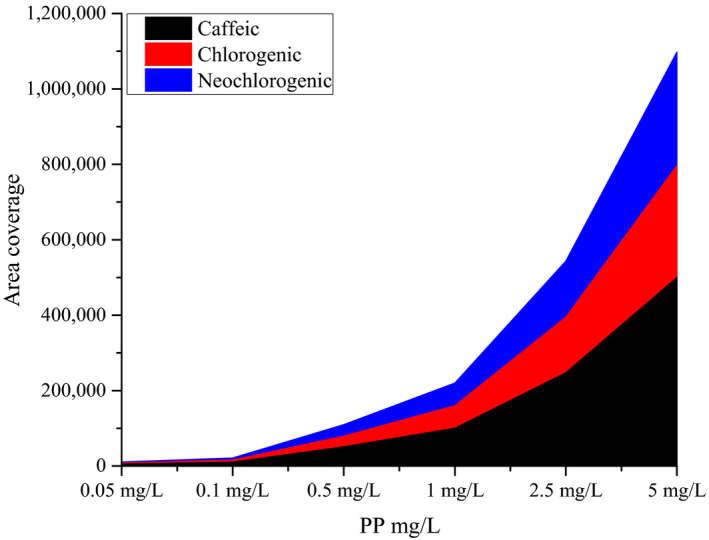
Peak height HPLC analysis of potato peel extract phenolic compounds

Moreover, HPLC, finding revealed that *caffeic, chlorogenic,* and *neochlorogenic* acids were the main bioactive compounds with a concentration of 374.08, 339.96, and 22.52 mg/L, respectively. Figure 1 showed that as the concentration of the potato peel in the solvent increase the content of caffeic, chlorogenic, and neochlorogenic content increase the impact of this was shown in MLC that the lowest potato peel concentration has lost its ability to create a zone of inhibition. This result is a similarity with the results of previous research work of Snchez (2014) says that *neochlorogenic* and *chlorogenic acid* together accounts 75% and 69% in the crude extract of phenolic fraction is accounts caffeic acid.

Plants extracts generally assumed to have antimicrobial compounds. The phenols are accountable for the antimicrobial ability of plant extract. These compounds used for self‐defend from the natural enemy attack that could help for resistibility of microbial infections (Lin, Labbe, & Shetty, 2004; Wojtaszek, 1997). The phenolic compounds found in PP extract which accounts 60%–80% are *neochlorogenic* acid, *chlorogenic* acid, and *caffeic* acid (Sánchez Maldonado, Mudge, Gänzle, & Schieber, 2014). The product of *chlorogenic* acid hydrolysis, *caffeic* acid has demonstrated for its substantial antimicrobial activity (Sánchez‐Maldonado, Schieber, & Gänzle, 2011). So these prominent phenolic compounds are responsible for the antimicrobial activity revealed in the study.

The difference between gram‐positive and gram‐negative bacteria to antimicrobial response differs in cell wall structure. The cell wall of gram‐positive contains single‐coat, whereas gram‐negative has multilayered structure bounded by external cell membrane (French, Whistler, Bemiller, & Paschall, 1984). Therefore, intra‐cellular spaces of bacterial can easily hyperacidified, triggering functional disorder of bacterial energy metabolism. However, the exterior lipopolysaccharide coating gives more buffering capacity to gram‐negative bacteria, functioning as a defensive fence for hydrophobic compounds (Haahn, Jones, Akha, & Rockland, 1977). Accordingly, the bacteria show less sensitivity to the antimicrobial activities of polyphenols. Thus, most plant sources antimicrobial have harmless to use in food. Therefore, PP extracts could be an alternative antimicrobial and promote the reduction in the use of synthetic preservatives in food.

### 3.2 Antioxidant potential of active edible film

#### 3.2.1 Morphological analysis of active film

**Figure 2:**
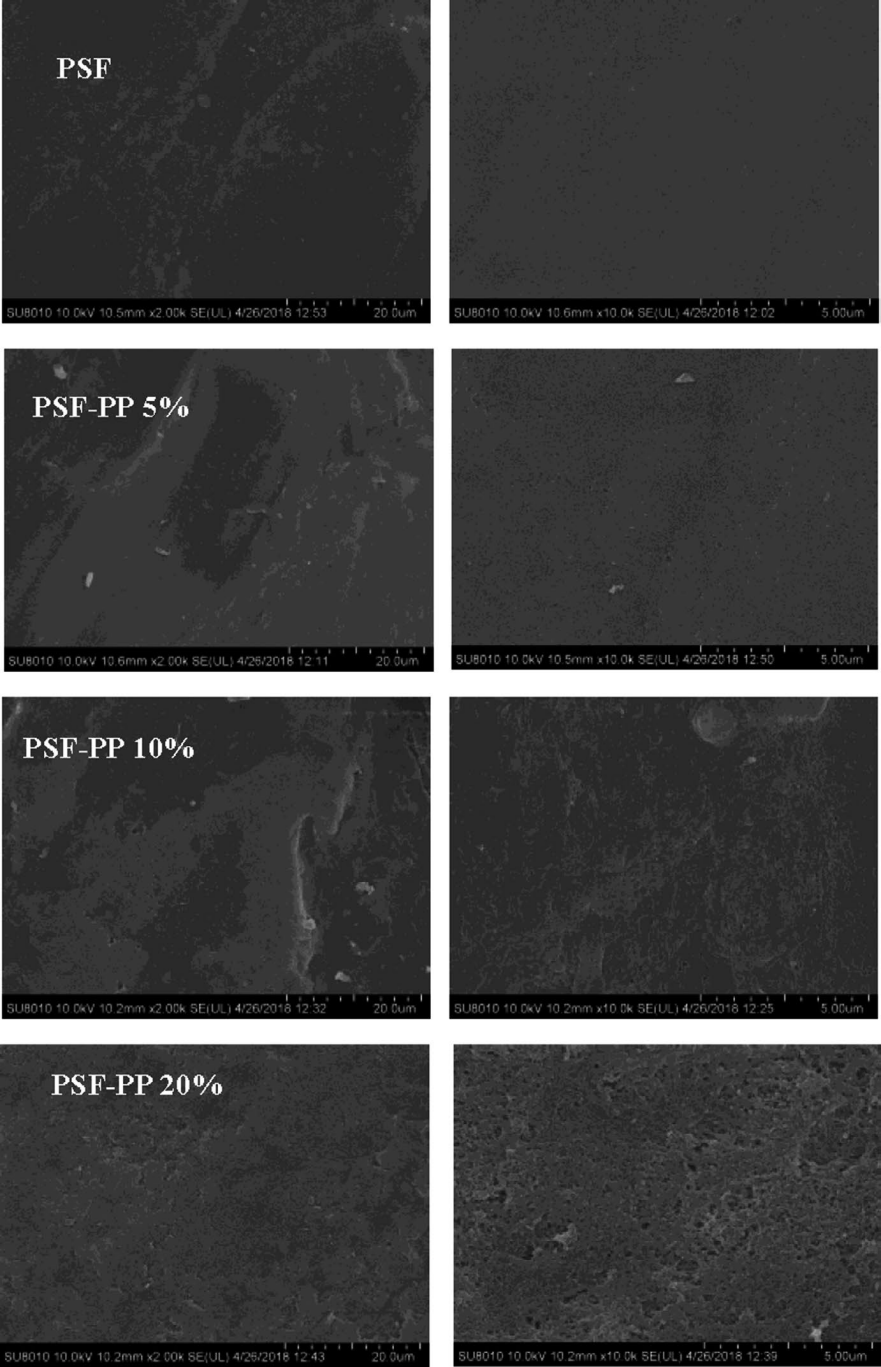
Micrographs obtained by scanning electron microscope cross sections of potato starch film

Figure 2 shows SEM micrographs of control (PSF), active film (PSF‐PPE 5%, PSF‐PPE 10%, and PSF‐PPE 20%). PSF film without potato peel extracts considered as control showed homogeneous surface structure, while in the active film some cracks were observed. As the quantity of potato peel aqueous increased in the film composition, the cracks over the film surface seen dispersed.

This could be because starch film has comparatively smooth surface impermeable for large molecules (French et al., 1984). When granules put in warm water, it initiates to hydrate then swell (Haahn et al., 1977). The multipart nature of the heated granule structure and the starch chains become more mobile as hydrogen bonding between adjacent glucose units is interrupted and water enters the matrix (Rodríguez‐rojo, Visentin, Maestri, & Cocero, 2012). The increase in chain mobility allows the starch film to become more flexible and swell. At the same time, amylose and small amounts of amylopectin begin to leach from the granule matrix into the aqueous medium (Shamekh, Forssell, Suortti, Autio, & Poutanen, 1999). Granule hydration further accelerated by the presence of small pores that reportedly span from the granule surface to the core region (Kim & Huber, 2008). Therefore, the change in structural showed in PSF and active PSF is the difference in the amount of aqueous PP with the starch film. So, farther research should focus on determining the optimal level of aqueous potato peel to minimize cracked and massive pore in the surface of the edible plastic film for better performance to be used as food packaging.

#### 3.2.2 TPC and antioxidant activity of active film

The TPC, DPPH inhibition %, and migration of polyphenols of the active film are shown in Table 3; the phenol content and DPPH‐scavenging activity of active film showed that increasing amount of PPE to the formulations will produce higher polyphenol amount in the active film. The DPPH‐radical scavenging activities active film increase gradually when increased the phenol content in samples. The potato starch film antioxidant content tailored in the film. The similar result is shown in earlier research work done by Pieros‐Hernandez (2017).

**Table 3:** TPC, DPPH‐scavenging activity, and polyphenol migration test of active film

Film	mg GAE/g dried film	Inhibition %	Polyphenol migration test	
			Food simulants	mg GAE/kg of simulant
PSF‐PP5%	10.34 ± 2.71	22.40 ± 1.2	Water	56 ± 2.3
			Ethanol	34 ± 2.1
PSF‐PP10%	15.21 ± 0.26	34.52 ± 0.1	Water	134 ± 1.3
			Ethanol	42 ± 3.1
PSF‐PP20%	22.05 ± 1.03	55.12 ± 2.2	Water	246 ± 4.2
			Ethanol	45 ± 2.1

Abbreviations: PP, potato peel; PSF, potato starch film.

**Figure 3:**
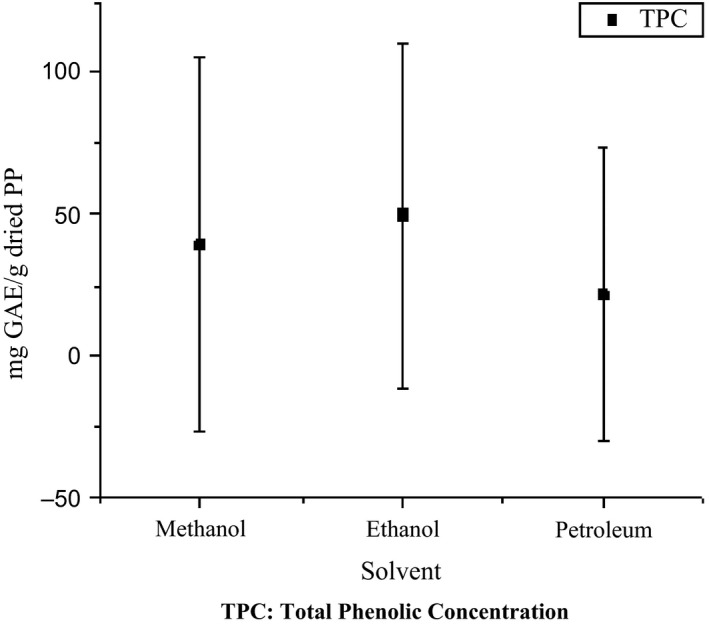
The total phenolic content of potato peel extract in different solvents

In Figure 3, the TPC of PP extracts of three solvents showed significant variation with (*p* < 0.05) value. The highest TPC attained from *ethanol* extracts (39 ± 0 mg of GAE/g) followed by *Methanol* (49 ± 2mg of GAE/g) and *Petroleum* (22 ± 1 mg of GAE/g of dried PP). The DPPH‐scavenging ability in between the solvents does not show a significant difference with % inhibition obtained 65 ± 1, 60 ± 2 and 51 ± 2 for *Methanol Ethanol* and *Petroleum*, respectively. On the contrary, TPC reported by Arun et al. (2015). The higher TPC content obtained from ethyl‐acetate extract (83.2 *GAE/g* of dried PP weight of extracts) and showed higher DPPH‐scavenging activity.

#### 3.2.3 Migration test of PPE from active film to food simulants

The polyphenol migration test using food simulant of water and 95% ethanol migrated from the active film to stimulants measured after seven days of the active film exposure inside the simulant (Table 3). The highest amounts and fast flow of polyphenols revealed in the active film the aqueous medium. In contrast to this, in the ethanol medium, the polyphenol amount was low. Active agents of a film release to any type of food from aqueous to fatty products, so the film could be used in active packaging (Yang et al., 2016). The migration of chemical compounds from active film packaging to food simulants can be affected by temperature, concentration, and the affinity of the compounds to the food simulant (Santos, Silva, & Andrade, 2017). In addition several factors influence migration of polyphenols such as nature of the bioactive compound, the chemical composition the reaction in‐between film and bioactive compound, the structural matrix, and the medium condition (Baner et al., 1992).

Similar results reported by Chang‐bravo, López‐córdoba, & Martino, 2014 says the quick diffusion of bioactive compounds from the film to the aqueous (simulants) could attribute water penetration into potato starch–glycerol matrix. Starch matrices are less swellable in the ethanol medium, and little quantity of the simulant reached penetration of the film matrix. This behavior is useful for applications for longer time release of antioxidants required.

## 4 CONCLUSIONS

Potato peel extract has antibacterial and antioxidant properties and active starch film through incorporating potato peel extracts was successfully developed. The phenolic compound in the extract has an ability to hinder fat oxidation and bacterial inhibition. The harmless nature of most plant extracts such as potato peel has more acceptability by food processing companies to use as a food preservative. Potato peel extracts can be used in different mechanisms for food preservative but incorporating with the starch film is the best alternative and run with the current global technology demanded environmentally friendly packaging product. Therefore, further studies addressing how to apply as a packaging system for food industries should be done.

## CONFLICT OF INTEREST

The authors declare that we do not have any conflict of interest.

## DECLARATION FOR HUMAN SUBJECTS

This study does not involve any human or animal testing.

## ETHICAL APPROVAL

The study's protocols and procedures were ethically reviewed and approved by Institute of Food and Science Technology, CAAS.
